# Brain Injury Markers: Where are We?

**DOI:** 10.3389/fneur.2014.00145

**Published:** 2014-08-04

**Authors:** Stefania Mondello, Frank C. Tortella

**Affiliations:** ^1^Department of Neurosciences, University of Messina, Messina, Italy; ^2^Brain Trauma Neuroprotection and Neurorestoration Branch, Walter Reed Army Institute of Research, Silver Spring, MA, USA

**Keywords:** biomarker, brain injury, traumatic brain injury, discovery, clinical practice

Traumatic brain injury (TBI), a growing public health problem, appears to result not only from major andprimary injury but also from a complex interplay among inflammatory, biochemical, and neurohormonal changes, as well as genetic components acting on brain tissue. As a result, characterization and classification of TBI requires multidimensional approaches that are able to encompass the diverse and highly complex clinical picture of TBI across the continuum of severities and broad spectrum of pathobiological processes. Emerging evidence suggests that an increasing number of biologic substances, commonly referred to in today’s vernacular as biomarkers, can provide unprecedented opportunities for detecting and classifying injury, and identifying pathophysiologic mechanisms potentially leading to more effective targeted therapies.

In this Research Topic, we include comprehensive reviews of the current literature on this topic ranging from proteomics techniques applied for the first time to central nervous system (CNS) biomarker discovery (1) to potential clinical applications of existing biomarkers of brain injury in specific settings such as ICU, pediatric TBI (2), and the military-relevant battlefield casualty (3). In particular, to address the unique circumstances and consequences of sustaining a TBI in combat and the demand for specific practices of management and care of soldiers, presentations (3, 4) have been included from outstanding researchers of the Combat Casualty Care Research Program (CCCRP) for Brain Trauma and Neuroprotection, a program specifically focused on developing neuroprotective and neurorestorative strategies for military-relevant TBI. We have also added a chapter on blast TBI to emphasize the potential problem of TBI following exposure to blast (5). Finally, we expanded discussions to explore the potential of brain damage biomarkers as tools for predicting long-term consequences of TBI (6) and to outline their roles in other CNS diseases such as neurodegeneration (Parkinson’s disease) (7), subarachnoid hemorrhage (8), and hypoxic ischemic encephalopathy (9, 10).

We have strived to assemble a multidisciplinary group of internationally recognized researchers and clinicians highly relevant to this research domain (11–13). As the translation of brain damage biomarkers has already transformed from research tools to being aids in clinical decision-making, this Research Topic will be evolutionary reading for neurotrauma scientists and clinicians interested in the potential of a simple biofluid-based diagnostic test to refine the clinical characterization of TBI offering more accurate disease phenotyping. Such improved molecular characterization integrated with traditional approaches, including clinical examination and structural and functional neuroimaging, will allow the field to develop improved clinical practice guidelines and tailor therapeutic interventions to the patient’s individual pathophysiology, thereby leading to effective management and improved patient outcome.

This Research Topic would not have been possible without the support and help of many people. First, we thank the chapter authors for devoting their time and effort to produce valuable contributions that provide comprehensive frameworks and critical insights. We also thank the members of the editorial board for their dedicated assistance and for providing informed perspectives on the chapters. Last, and most important, we thank all patients with TBI and their families for their invaluable contributions. To improve their outcome and quality of life represents our ultimate goal and our greatest source of inspiration to foster knowledge in this critical research area.

## Author Note

Material has been reviewed by the Walter Reed Army Institute of Research. There is no objection to its presentation and/or publication. The opinions or assertions contained herein are the private views of the authors, and are not to be construed as official, or as reflecting true views of Department of the Army or Department of Defense.

## Conflict of Interest Statement

The authors declare that the research was conducted in the absence of any commercial or financial relationships that could be construed as a potential conflict of interest.
